# *MAT* Loci Play Crucial Roles in Sexual Development but Are Dispensable for Asexual Reproduction and Pathogenicity in Rice Blast Fungus *Magnaporthe oryzae*

**DOI:** 10.3390/jof7100858

**Published:** 2021-10-13

**Authors:** Jiao-yu Wang, Shi-zhen Wang, Zhen Zhang, Zhong-na Hao, Xiao-xiao Shi, Ling Li, Xue-ming Zhu, Hai-ping Qiu, Rong-yao Chai, Yan-li Wang, Lin Li, Xiao-hong Liu, Xiao-xiao Feng, Guo-chang Sun, Fu-cheng Lin

**Affiliations:** 1 State Key Laboratory for Managing Biotic and Chemical Treats to the Quality and Safety of Agro-Products, Institute of Plant Protection and Microbiology, Zhejiang Academy of Agricultural Sciences, Hangzhou 310021, China; wangjiaoyu78@sina.com (J.-y.W.); wsz_1231@outlook.com (S.-z.W.); zzhangcn928@sina.com (Z.Z.); haozhongna1999@sina.com (Z.-n.H.); 15268853841@163.com (X.-x.S.); 21516131@zju.edu.cn (X.-m.Z.); qiuhping@163.com (H.-p.Q.); rychai@sina.com (R.-y.C.); ylwang88@aliyun.com (Y.-l.W.); 21616143@zju.edu.cn (L.L.); 2 School of Agricultural and Food Sciences, Zhejiang Agriculture and Forest University, Hangzhou 311300, China; liling-06@163.com; 3 Institute of Biotechnology, Zhejiang University, Hangzhou 310058, China; xhliu@zju.edu.cn; 4 Agricultural Experiment Station, Zhejiang University, Hangzhou 310058, China; xxvon@zju.edu.cn

**Keywords:** *MAT* loci, *MAT* genes, sexual reproduction, pathogenicity, *Magnaporthe oryzae*

## Abstract

*Magnaporthe oryzae*, a fungal pathogen that causes rice blast, which is the most destructive disease of rice worldwide, has the potential to perform both asexual and sexual reproduction. *MAT* loci, consisting of *MAT* genes, were deemed to determine the mating types of *M. oryzae* strains. However, investigation was rarely performed on the development and molecular mechanisms of the sexual reproduction of the fungus. In the present work, we analyzed the roles of two *MAT* loci and five individual *MAT* genes in the sex determination, sexual development and pathogenicity of *M. oryzae*. Both of the *MAT1-1* and *MAT1-2* loci are required for sex determination and the development of sexual structures. *MAT1-1-1*, *MAT1-1-3* and *MAT1-2-1* genes are crucial for the formation of perithecium. *MAT1-1-2* impacts the generation of asci and ascospores, while *MAT1-2-2* is dispensable for sexual development. A GFP fusion experiment indicated that the protein of *MAT1-1-3* is distributed in the nucleus. However, all of the *MAT* loci or *MAT* genes are dispensable for vegetative growth, asexual reproduction, pathogenicity and pathogenicity-related developments of the fungus, suggesting that sexual reproduction is regulated relatively independently in the development of the fungus. The data and methods of this work may be helpful to further understand the life cycle and the variation of the fungus.

## 1. Introduction

Sexual reproduction is one of the key events in the evolution of life, as it provides more abundant genetic variation and progeny diversity, which helps organisms survive and adapt to changing environments [1]. Compared to asexual reproduction, sexual reproduction involves more complex processes, such as plasmogamy, karyogamy and meiosis [2].

Fungi are composed of organisms belonging to more than 120,000 species, ranging from yeasts to higher mushrooms, and among them, many economically important species are used in industrial production and the food industry or for their ability to cause destructive diseases in plants. Reproduction in fungal species involves sexual and asexual processes, and in many cases, the two processes coexist in one species, making fungi a good model for understanding sexual evolution. The sexual reproduction of fungal species includes two types, homothallism and heterothallism. Homothallic fungal species are capable of self-mating, whereas heterothallic ones require compatible partners with opposite mating types to complete the mating and sexual reproductive process.

The gene loci determining the mating types are designated as *MAT* loci, generally as *MAT1-1* and *MAT1-2* in the heterothallic species, which represent the opposite mating types [3]. A heterothallic strain only encounters strains with the opposite mating type, and then fertilization can occur [4]. Each of the *MAT* loci usually contains several genes. Although *MAT* genes are factors that promote sexual development and determine the reproductive mode [5], their biological functions remain largely undefined and vary greatly in different species [6,7].

*Magnaporthe oryzae* (syn. *Pyricularia oryzae*) causes rice blast, the most destructive disease of rice, and is ranked top of the list of the top 10 fungal pathogens [8]. The fungus has evolved diverse races and pathotypes that infect various rice cultivars and other gramineous crops and weeds, such as wheat, barley and ryegrass. *M. oryzae* is a heterothallic ascomycete and possesses the ability of both asexual and sexual reproduction [9]. In rice fields, the spread and host infection of the fungus is accomplished by asexual spores. While in the lab, when two proper strains of opposite mating types are co-cultured, sexual reproduction can occur, forming sexual structures, such as perithecia, asci and ascospores [10].

In the past three decades, the molecular basis of the pathogenicity of *M. oryzae* has been investigated intensively. Hundreds of genes involved in infection and asexual reproduction have been identified and functionally characterized [11,12,13]. To date, the sexual reproduction of the fungus has still not been directly found in its natural environment. Despite this, the population structures and the predominant races of *M. oryzae* in the field change rapidly, which makes rice blast disease difficult to efficiently control. Such a rapid variation of the fungus hints at the possible existence of natural sexual reproduction. Moreover, studies on population genomics in recent years support this speculation [14,15]. An investigation of the sexual mechanism of *M. oryzae* is thus significant for a better understanding of whether and how sexual reproduction contributes to genetic diversity and pathogenicity variation. However, the molecular mechanisms of sexual reproduction of the fungus remain largely unknown, and the functions of the *MAT* genes have not yet been characterized by direct molecular strategies.

In the present work, we investigated the functions of the *MAT* loci and *MAT* genes in sexual development and the related processes using gene knockout strategies in *M. oryzae*. To our knowledge, this is the first direct investigation of the sexual processes at the molecular level in the fungus. The resulting data and strains may be helpful to further understand the life cycle and the variation of the fungus.

## 2. Materials and Methods

### 2.1. Strains and Culture

*M. oryzae* strains Guy11 (*MAT1-1*), TH16 (*MAT1-1*), 2539 (*MAT1-2*), 70-15 (*MAT1-2*) and TH3 (*MAT1-2*) were used as wild-type strains and cultured using routine methods. Complete medium (CM) was used to culture the strains for vegetative growth and asexual processes.

### 2.2. Sexual Reproduction

The *M. oryzae* strains Guy11, TH16, 2539, 70-15 and TH3 were cross-cultured on oatmeal medium (OMA), pairwise according to opposite mating types, to induce sexual reproduction. The OMA cultures were first incubated at 28 °C with 12/12 h light–dark cycles for 7 days and then moved to 22 °C with full illumination for 15 days. The experiments were repeated 3 times, and 3 replicates were performed for each group.

### 2.3. MAT Loci Deletion and Mutant Recovery

*MAT1-1* and *MAT1-2* loci were deleted by homologous recombination strategies. *MAT1-1* was deleted in Guy11 and TH16, whereas *MAT1-2* was deleted in 70-15, 2539 and TH3. The up- and down-stream fragments of *MAT1-1* and *MAT1-2* were amplified using the DNA sample from Guy11 or 70-15 as a template and ligated into pKO-Hph [16] by *Pst I/BamH I* and *EcoR I/Xho I* digestion, respectively, to generate two knockout vectors, pKO-MAT1-1 and pKO-MAT1-2. Then, pKO-MAT1-1 and pKO-MAT1-2 were introduced into the proper *M. oryzae* strains via *Agrobacterium tumefaciens*-mediated transformation (*At*MT) to induce the targeted replacement of *MAT1-1* or *MAT1-2* loci with a hygromycin resistance gene (*Hph*), generating the *MAT* loci deletion mutants Guy11*^Δmat1^*, 70-15*^Δmat2^*, 2539*^Δmat2^* and TH3*^Δmat2^*.

For mutant recovery, the fragments covering the full length of the *MAT* loci were amplified and inserted into p1300-Bar (Li et al. 2014) by *Pst I/BamH I* digestion to generate the complementary vectors p1300-MAT1-1 and pl300-MAT1-2. The vectors were integrated into the corresponding mutants using *At*MT to generate the *MAT* loci recovery strains Guy11*^Δmat1/MAT1^*, 70-15*^Δmat2/MAT2^*, 2539*^Δmat2/MAT2^* and TH3*^Δmat2MAT2^*.

### 2.4. MAT Gene Deletion and Mutant Recovery

For *MAT* gene deletion, the up- and down-stream fragments of each *MAT* gene were amplified and inserted into pKO-Hph (Li et al. 2014) via *Pst I/BamH I*, *EcoR I/Xho I*, *Hind III/BamH I*, *Sma I/Kpn I* and *Sma I/Xho I* digestion to generate the gene replacement vectors pKO-MAT1-1-1, pKO-MAT1-1-2, pKO-MAT1-1-3, pKO-MAT1-2-1 and pKO-MAT1-2-2, respectively. The vectors were introduced into the proper *M. oryzae* strains via *At*MT to induce the targeted replacement of the *MAT* genes with the *Hph* gene, generating the mutants Guy11*^Δ111^*, Guy11*^Δ112^*, Guy11*^Δ113^*, 70-15*^Δ121^*, 70-15*^Δ122^*, 2539*^Δ122^* and TH3*^Δ122^*.

For mutant recovery, the fragments containing the full length of the individual *MAT* genes were amplified and inserted into p1300-Bar via *Pst I/BamH I* digestion to generate the complementary vectors p1300-MAT1-1-1, p1300-MAT1-1-2, p1300-MAT1-1-3, p1300-MAT1-2-1 and p1300-MAT1-2-2. The vectors were integrated into the corresponding mutant strains via *At*MT, generating the recovery strains Guy11*^Δ111/111^*, Guy11*^Δ112/112^*, Guy11*^Δ113/113^*, 70-15*^Δ121/121^*, 70-15*^Δ122/122^*, 2539*^Δ122/122^* and TH3*^Δ122/122^*.

Genomic PCR was used to identify and confirm the recombinant events by applying targeted genes, the *Hph* gene and up- and down-stream fragments.

### 2.5. RNA Extraction and qRT-PCR

The wild-type strains, Guy11, 70-15, TH3 and TH16, and the mutant strains, Guy11*^Δ111^*, Guy11*^Δ112^*, Guy11*^Δ113^*, 70-15*^Δ121^* and 70-15*^Δ122^*, were incubated separately or pairwise according to opposite mating types on OMA. The RNA extraction and cDNA synthesis were performed using a reagent kit (TIANGEN, Beijing, China), following the manufacturer’s instructions. The RT-qPCR experiments were performed with the cDNA samples as templates by following the manual of the reagent kit (Roche, Basel, Switzerland). The relative transcription levels were calculated with the β-tubulin gene (MGG_00604) as a reference. For each experiment, at least three independent biological replicates were conducted and statistically analyzed.

All of the primers used for vector construction, the confirmation of mutants and recovery strains and qRT-PCR are listed in Table S1.

### 2.6. Microscopic Analysis

The sexual structures were observed under a light microscope Axio Imager A2 ( Zeiss, Jena, Germany) and a stereo microscope SZX2-ILLT (Olympus, Tokyo, Japan). The structures were also paraffin sectioned or stained with calcofluor white (50 μg/mL) and detected under a fluorescence microscope Axio Imager A2 (Zeiss, Jena, Germany) or a laser scanning confocal fluorescence microscope LSM880 (Zeiss, Jena, Germany). Cryoelectronic scanning electron microscopy Regulus 8100 (Hitachi, Tokyo, Japan) and transmission electron microscopy H7650 (Hitachi, Tokyo, Japan) were also used to analyze the ultrastructure of the sexual offspring.

### 2.7. Subcellular Distribution

To monitor the subcellular distribution of the *MAT* genes in *M. oryzae*, the coding sequences of the *MAT* genes were amplified and introduced into the vector p1300BMG-C [16] by *Sma I/EcoR I* digestion to generate pBMG-111, pBMG-112, pBMG-113, pBMG-121 and pBMG-122. The vectors were introduced into the wild-type strain 70-15 via *At*MT, resulting in the fluorescent transformants 70-15*^PBMG-111^*, 70-15*^PBMG-112^*, 70-15*^PBMG-113^*, 70-15*^PBMG-121^* and 70-15*^PBMG-122^*, which were selected and confirmed by genomic PCR and GFP fluorescence detection. The fluorescence of the strains was detected under a fluorescence microscope.

### 2.8. Assays for Asexual Reproduction, Carbon Utilization, Chemical Resistance and Pathogenicity-Related Developments

The experiments to assay vegetative growth, asexual reproduction, carbon utilization, chemical resistance, host inoculation and pathogenicity-related developments were all performed using typical methods as described previously [16].

## 3. Results

### 3.1. Both of the MAT Loci Are Indispensable for Sexual Reproduction

**Figure 1:**
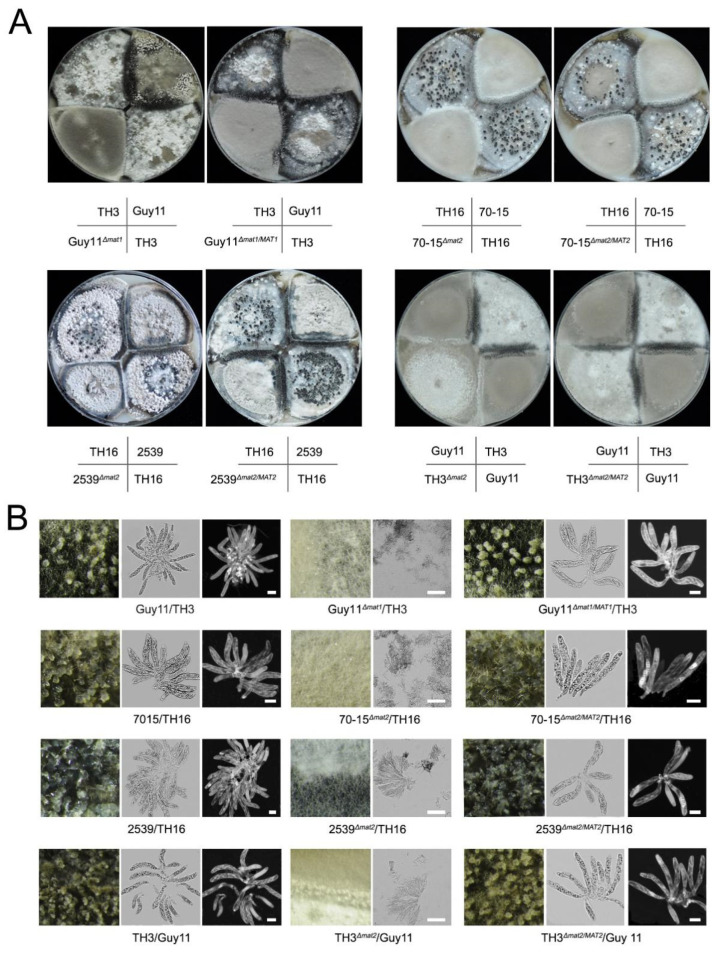
Characterizing the function of *MAT* loci in sexual development by the gene deletion strategy. (**A**) Comparison of the development of perithecia of wild-type, *MAT* loci mutants and recovery strains by crossing with their partner strains on OMA plates; (**B**) the perithecia generated in different combinations were observed in 60× magnification under a stereomicroscope, and the asci were stained with calcofluor white and detected under a fluorescence microscope. Bars = 20 μm.

The functions of the *MAT1-1* and *MAT1-2* loci (abbr. *MAT1* and *MAT2*) in the sexual development of *M. oryzae* were investigated via gene knockout strategies. Mutants lacking *MAT1-1* were derived from Guy11, and those lacking *MAT1-2* were derived from 70-15, 2539 and TH3. Then, the sexual development ability of the mutants was tested by crossing experiments. The combinations of the wild-type strains could generate asci and ascospores normally (Figure 1; Table 1). When the *Δmat1* and *Δmat2* strains were crossed with their corresponding wild-type strains, such as Guy11*^Δmat1^* with *TH3*, 70-15*^Δmat2^* with *TH16*, 2539*^Δmat2^* with TH16 and TH3*^Δmat2^* with Guy11, the sexual structure was hardly formed. In addition, the recovery strains Guy11*^Δmat1/MAT1^*, 70-15 *^Δmat2/MAT2^*, 2539*^Δmat2/MAT2^* and TH3*^Δmat2/MAT2^* regained the ability to form sexual structures when mated with the strains with the opposite mating type. Moreover, the morphology of the perithecia and asci that were formed by the recovery strains showed no difference compared to those by the wild-type combinations (Figure 1). The data indicated that the *MAT* loci were indispensable for perithecium formation and sexual development.

### 3.2. MAT1-1-2 and MAT1-1-3 Genes Are Up-Regulated during Sexual Reproduction

**Figure 2:**
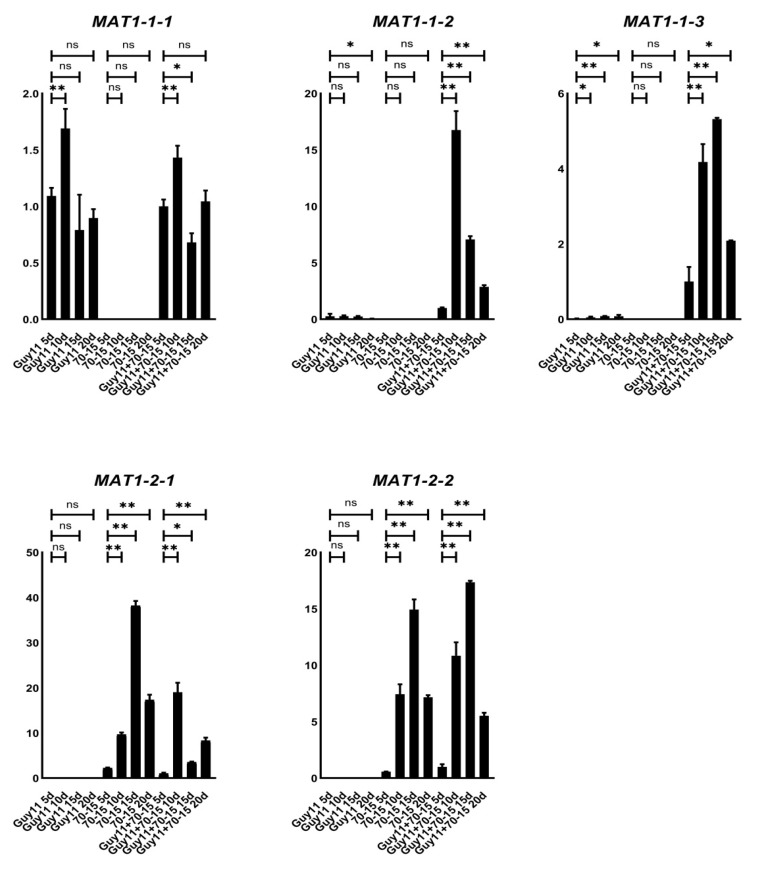
Transcription levels of each individual *MAT* gene. The relative transcription levels of each individual *MAT* gene in wild-type strains (70-15 and Guy11) cultured alone and during the sexual process (70-15 crossed with Guy11) were tested using qRT-PCR at 5 10, 15 and 20 dpc (days post-cultivation). The expression levels in Guy11 strain cultured alone on OMA were used as control. Mean and standard error were calculated from three independent biological replicates. The difference significance for each gene in the different samples was calculated by comparing the genes in the cross-cultured samples (Guy11 + 70-15) at 5 dpc. Single stars indicate the significance at the 0.5 level and double stars indicate it at the 0.01 level.

To clarify the expression patterns of the *MAT* genes during the mating process, we cross-cultured the wild-type strains Guy11 and 70-15 on OMA for 20 days and investigated the relative transcription levels of each *MAT* gene at 5, 10, 15 and 20 dpc (days post-cultivation). As predicted, the transcripts of *MAT1-1-1*, *MAT1-1-2* and *MAT1-1-3* could be detected only in the Guy11 strain, while *MAT1-2-1* and *MAT1-2-2* only in 70-15 (Figure 2). In cross-cultured Guy11/70-15, the transcription levels of *MAT1-1-2* and *MAT1-1-3* were found to be significantly increased compared to those in the separately cultured Guy11 strain, suggesting that the *MAT1-1-2* and *MAT1-1-3* genes were up-regulated during sexual reproduction. Meanwhile, the transcription levels of all *MAT* genes tended to peak at 10–15 dpc, the key period for the formation of perithecium; then, along with the development of the sexual processes, the transcription of the genes reduced gradually.

### 3.3. Subcellular Distribution of the MAT Proteins

**Figure 3:**
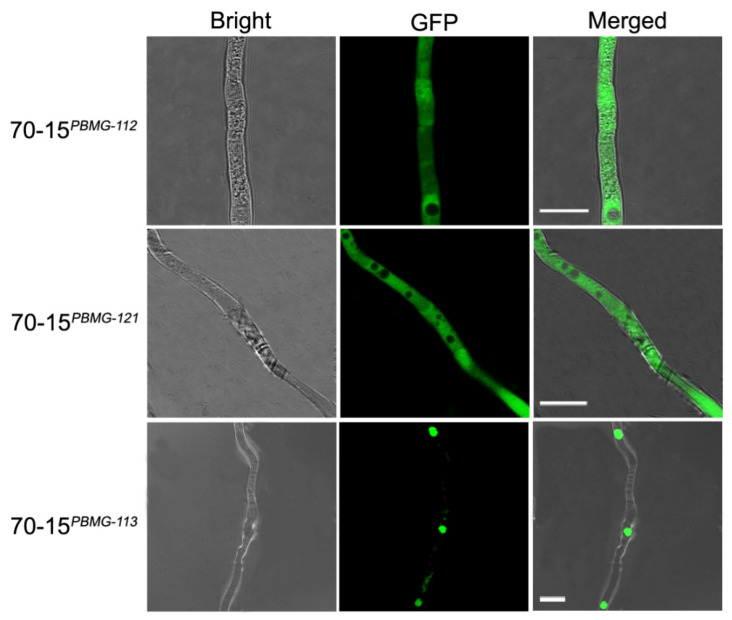
Subcellular distribution of the MAT proteins in 70-15 were detected using the GFP fusion method. The GFP fluorescence in hyphae of 70-15*^PBMG-112^*, 70-15*^PBMG-121^* and 70-15*^PBMG-113^* was observed under a fluorescence microscope. MAT1-1-3 is distributed in the nucleus while MAT1-1-2 and MAT1-2-1 are cytosolically distributed. Bars = 10 μm.

To elucidate the subcellular distribution of the *MAT* proteins, GFP-tagged versions of the *MAT* genes were constructed and introduced into *M. oryzae* strain 70-15, generating the fluorescent strains 70-15*^PBMG-112^*, 70-15*^PBMG-113^* and 70-15*^PBMG-121^*. The 70-15*^PBMG-113^* strain emitted fluorescence as a punctate pattern concentrated at the nucleic regions, indicating that MAT1-1-3 was distributed in the cell nucleus (Figure 3), corresponding with its potential as a transcription factor. However, the 70-15*^PBMG-112^* and 70-15*^PBMG-121^* strains emitted dispersive fluorescence in the hyphae, which suggested the cytoplasmic distribution of MAT1-1-2 and MAT1-2-1.

### 3.4. MAT Genes Contribute Differently to the Formation of Sexual Structures

**Figure 4:**
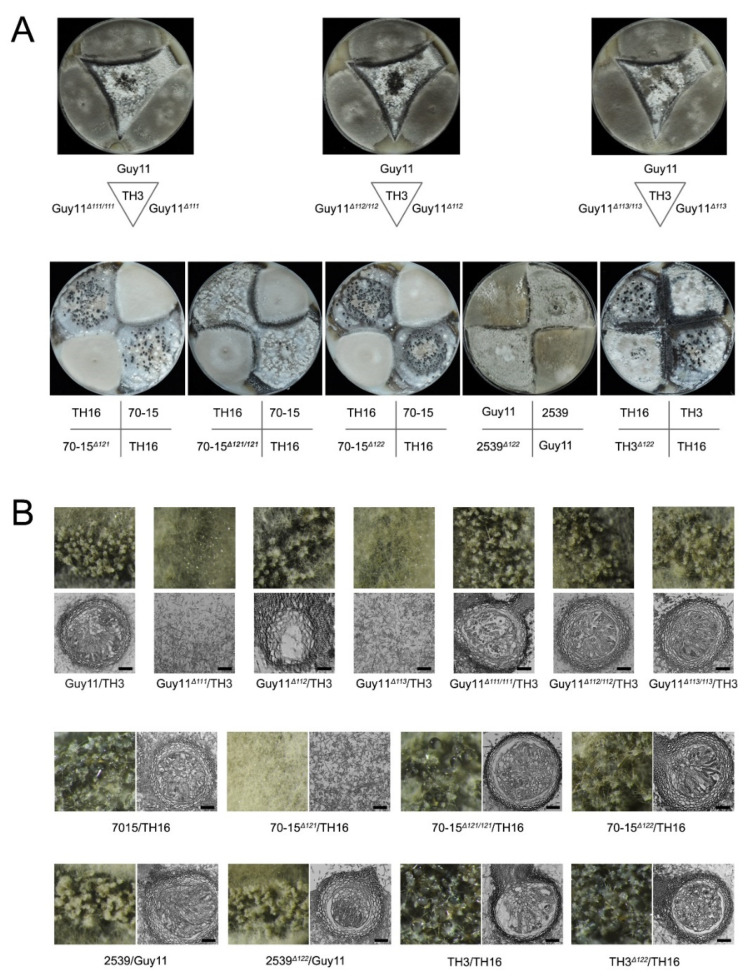
Function of individual *MAT* genes in sexual reproduction. (**A**) Crossing experiments of the wild-type strains, mutants and recovery transformants on OMA plates; (**B**) the perithecia generated in different combinations were observed in 60× magnification under a stereomicroscope, and the inner structures were observed via paraffin section method. Bars = 20 μm.

Although mutants lacking the whole *MAT* loci were unable to undergo sexual development, the roles of the individual genes in the *MAT* loci are undetermined. We thus generated mutants of each individual *MAT* gene. Each mutant, Guy11*^Δ111^*, Guy11*^Δ112^*, Guy11*^Δ113^*, 70-15*^Δ121^*, 70-15*^Δ122^*, 2539*^Δ122^* and TH3*^Δ122^*, was mated with their corresponding wild-type strains (Figure 4; Table 2). The mutants of Guy11*^Δ111^*, Guy11*^Δ113^* and 70-15*^Δ121^* lost the ability to produce perithecium with their corresponding wild-type strains of opposite mating types, indicating the deletion of any of the three genes leads to infertility of the fungus. Meanwhile, the complement strains, Guy11*^Δ111/111^*, Guy11*^Δ113/113^* and 70-15*^Δ121/121^,* fully recovered the ability to produce perithecium, confirming the vital roles of *MAT1-1-1*, *MAT1-1-3* and *MAT1-2-1* in perithecium formation. In contrast, the crossing of Guy11*^Δ112^*, 70-15*^Δ122^*, 2539*^Δ122^* and TH3*^Δ122^* with their corresponding wild-type strains exhibited no significant difference in sexual development compared with the combinations of the wild-type strains, indicating that *MAT1-1-2* and *MAT1-2-2* were dispensable for perithecium formation.

To further reveal the roles of the genes in sexual development, the structures of the perithecia, the asci and the ascospores were further investigated. The perithecia formed well in the cross of Guy11*^Δ112^* and TH3; however, no asci and ascospores could be found in these perithecia, indicating that the lack of *MAT1-1-2,* though did not interfere with the development of perithecium, hindered the generation of asci and ascospores (Figure 4B). In contrast, the mutants 70-15*^Δ122^*, 2539*^Δ122^*, and TH3*^Δ122^* developed asci and ascospores normally when crossed with the wild-type strains of the opposite mating type, indicating that *MAT1-2-2* is dispensable for the development of asci and ascospores. Meanwhile, the complement strains Guy11*^Δ111/111^*, Guy11*^Δ112/112^*, Guy11*^Δ113/113^* and 70-15*^Δ121/121^* fully regained the ability to produce normal asci and ascospores.

### 3.5. Deleting Any of the Individual MAT Genes Down-Regulated the Others

**Figure 5:**
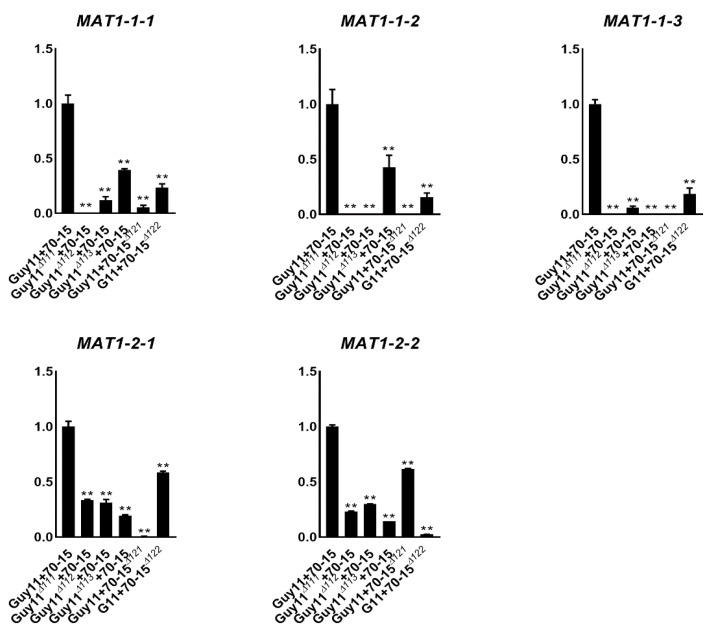
Transcription levels of the individual *MAT* genes in the *MAT* mutants. Relative transcription levels of the individual *MAT* genes in the crossing of Guy11 and 70-15, Guy11*^∆111^* and 70-15, Guy11*^∆112^* and 70-15, Guy11*^∆113^* and 70-15, Guy11 and 70-15*^∆121^* and Guy11 and 70-15*^∆122^*. The transcription levels in the wild-type crossing (Guy11 + 70-15) were used as control for each *MAT* gene. Mean and standard error were calculated from three independent biological replicates. Single stars indicate significance at the 0.5 level and double stars indicate it at the 0.01 level.

To investigate whether the expression of the *MAT* genes was inter-affected between each other, we investigated the relative transcription levels of the *MAT* genes in the mutants. Guy11*^Δ111^*, Guy11*^Δ112^*, Guy11*^Δ113^*and Guy11 were crossed with 70-15, 70-15*^Δ121^*and 70-15*^Δ122^*, respectively, and the relative transcription levels of the genes were assessed using qPCR. All of the five *MAT* genes were highly expressed in the wide-type cross, Guy11/70-15, while they were significantly reduced in the crosses containing any of the mutants (Figure 5). For example, *MAT1-1-1* was highly expressed in Guy11/70-15 and more down-regulated in the cross of Guy11*^Δ112^*/70-15, Guy11*^Δ113^*/70-15, Guy11/70-15*^Δ121^*and Guy11/70-15*^Δ122^*. The same situation was detected for *MAT1-1-2* and *MAT1-1-3*. These results suggest that the expression of *MAT1-1-1*, *MAT1-1-2* and *MAT1-1-3* was severely interfered with by other *MAT* genes. *MAT1-2-1* and *MAT1-2-2* were also down-regulated by the deletion of the other *MAT* genes but in milder levels than that of *MAT1-1-1*, *MAT1-1-2*, and *MAT1-1-3*. Moreover, the inter-effects between *MAT1-2-1* and *MAT1-2-2* were the smallest ones. The results of the transcription support the fact that somehow *MAT1-2-2* has less impact on sexual development than the other genes.

### 3.6. The MAT Loci Are not Required in Vegetative Growth, Asexual Reproduction or Pathogenicity

**Figure 6:**
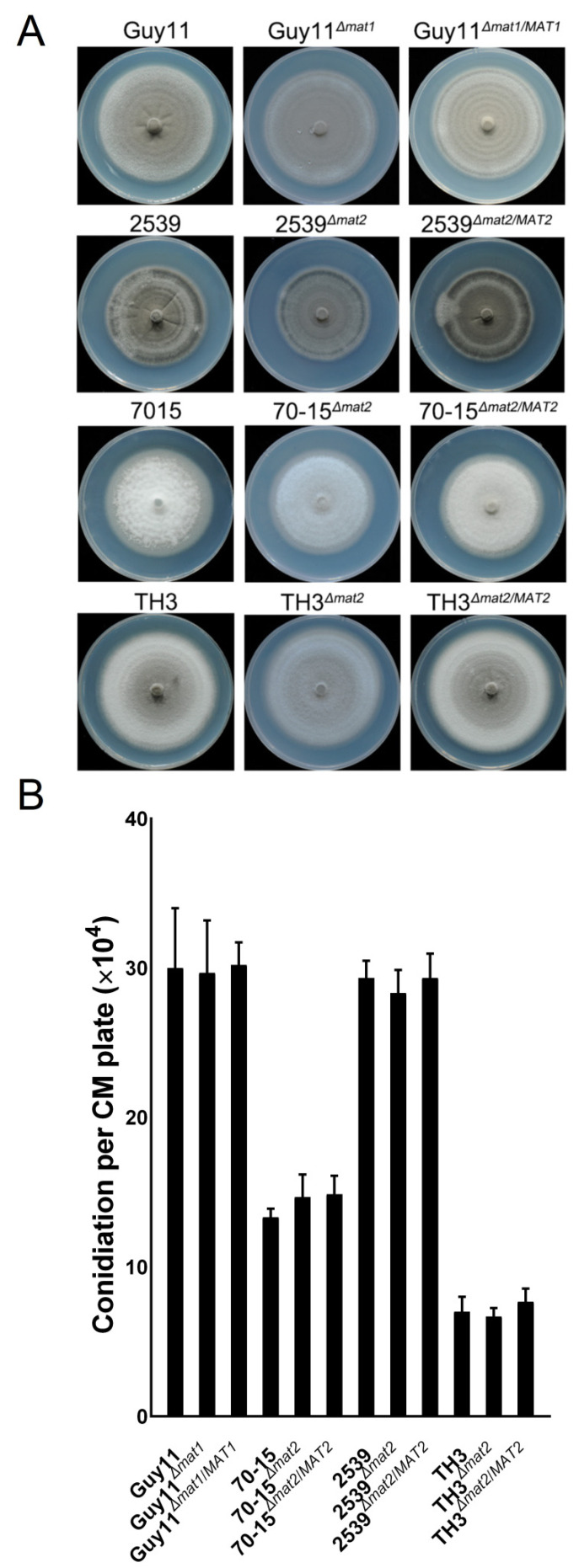
Colonial morphology and conidiation of the *MAT* loci mutants. (**A**) Colonial morphology of the mutants Guy11*^Δmat1^*, 2539*^Δmat2^*, 70-15*^Δmat2^* and TH3*^Δmat2^*, the wild-type strains Guy11, 2539, 70-15 and TH3, and the complement strains Guy11*^Δmat1/MAT1^*, 2539*^Δmat2/MAT2^*, 70-15*^Δmat2/MAT2^* and TH3*^Δmat2/MAT2^* cultured on CM for 7 days. (**B**) The conidiation ability of the strains was tested by counting the conidia harvested from 7-day colonies on CM. Mean and standard error were calculated from three independent biological replicates.

To clarify whether the *MAT* loci and *MAT* genes are involved in vegetative growth, asexual development or pathogenicity of the fungus, the mutants of the *MAT* loci and individual *MAT* genes were compared with the wild-type strains using routine procedures. Incubated on CM for 7 days, no significant differences of the mutants were found in the colony morphology and growth rates compared to the wild-type strains and the complement strains (Figure 6A and Figure S1). The ability of the *MAT* loci mutants to produce asexual conidia was not influenced either (Figure 6B). The data indicate that the *MAT* loci and *MAT* genes are dispensable for vegetative growth and asexual conidiation of the fungus.

**Figure 7:**
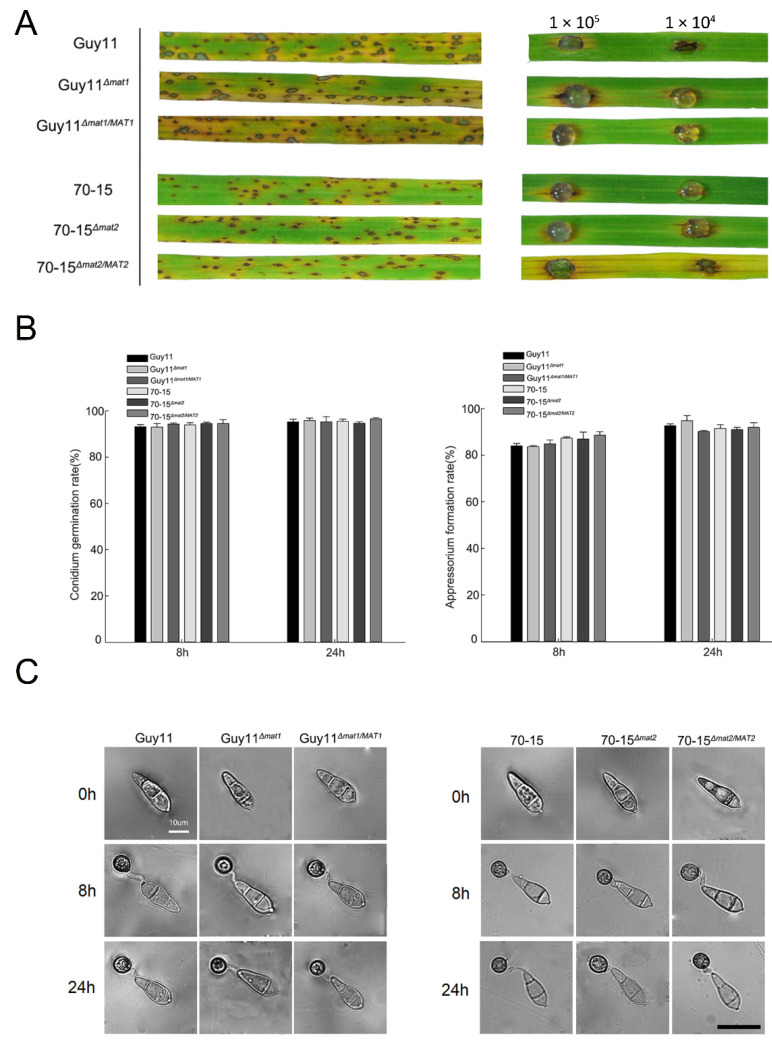
The pathogenicity and infection-related morphology were unaltered in *MAT* loci mutants. (**A**) The 2-week-old rice seedlings (**left**) were spray inoculated with the conidial suspensions at 1 × 10^5^ conidia/mL for Guy11 and Guy11-derived strains and 5 × 10^4^ conidia/mL for 70-15 and 70-15-derived strains; the detached rice leaves (**right**) were drop inoculated with the conidial suspensions at 1 × 10^5^ conidia/mL and 1 × 10^4^ conidia/mL. The symptoms were observed at 7 days post-inoculation. (**B**) The conidia of the strains were incubated on hydrophobic membranes to allow gemination and appressorium formation. The conidial germination rates (**left**) and appressorium formation rates (**right**) were calculated and compared at 0, 8 and 24 hpi (hours post-incubation). Mean and standard error were calculated from three independent biological replicates. (**C**) the morphology of the conidia and appressoria of the strains at 0, 8 and 24 hpi.

Inoculation was performed on rice seedlings and detached rice leaves with conidial suspensions of the wild-type strains Guy11 and 70-15, the mutants Guy11*^Δmat1^* and 70-15*^Δmat2^* and the complement strains Guy11*^Δmat1/MAT1^* and 70-15*^Δmat2/MAT2^*. The rice seedlings and the leaves inoculated with any of the strains generated typical blast lesions without any significant difference in symptoms (Figure 7A), indicating that the deletion of the *MAT* loci has no impact on the pathogenicity of *M. oryzae*. To further determine whether the *MAT* loci a play role in infection-related development, the conidia of the mutants were allowed to germinate and form appressoria. The rates of conidial germination and the appressorium formation of the mutants were equivalent to those of the wild-type strains, and the appressorial morphology was unaltered (Figure 7B,C), indicating that the *MAT* loci were irrelevant to these infection-related developments.

**Figure 8:**
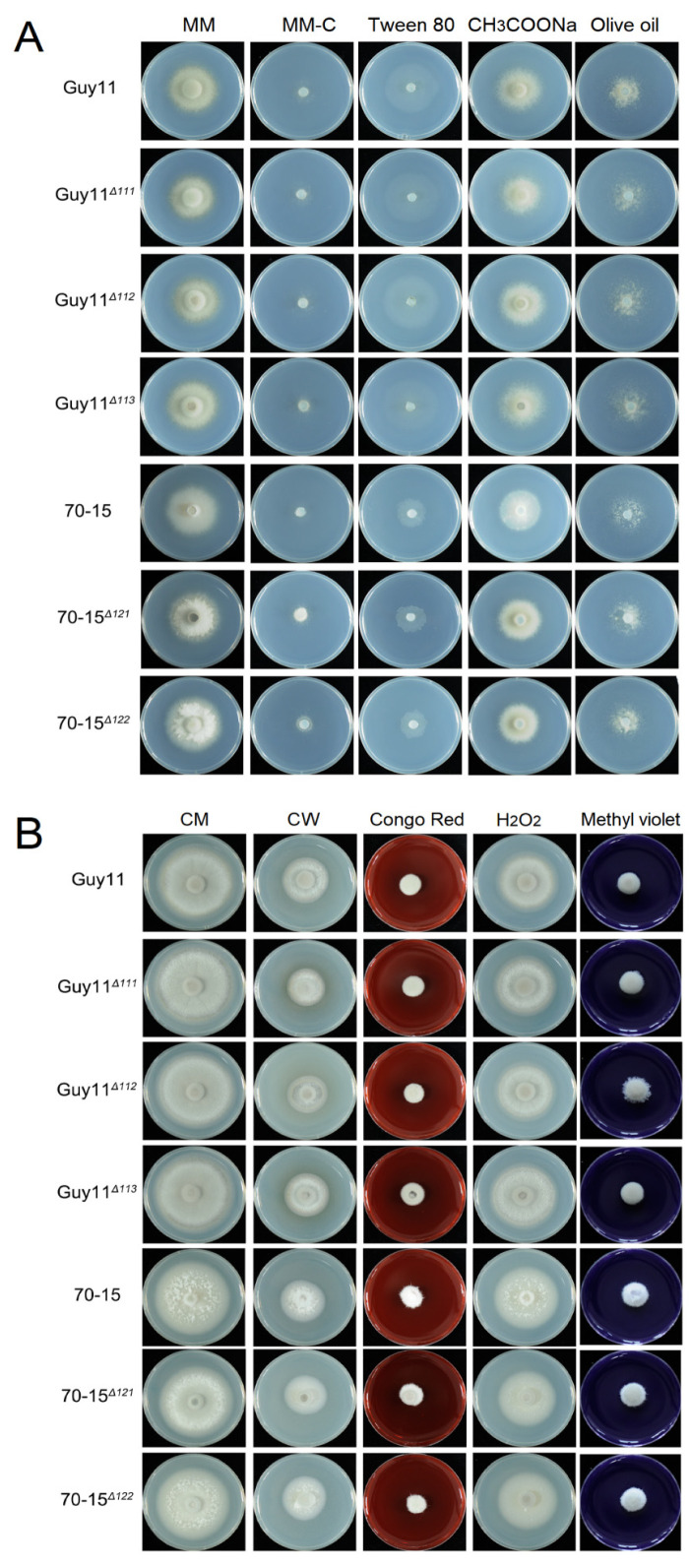
The carbon utilization, cell wall biogenesis and ROSs endurance were unaffected in the *MAT* gene deletion mutants. (**A**) The strains were cultured on MM; MM-C; and MM-C supplemented with 1% Tween 80, 50 mM CH_3_COONa or 1% Olive oil at 28 °C for 7 days. (**B**) The strains were cultured on CM and CM containing 150 μM calcofluor white (CW), 200 μM Congo red, 0.1% hydrogen peroxide or 4 mM Methyl violet at 28 °C for 7 days.

The carbon utilization of the *MAT* gene mutants was tested on CM, minimal medium (MM), MM lacking a carbon source (MM-C) and MM-C supplemented with various carbon sources (MM-C + 1% Tween 80, MM-C + 50 mM CH_3_COONa or MM-C + 1% olive oil). On these media, no significant difference in radical growth and colonial morphology was found between the wild-type and mutant strains, indicating that carbon utilization was not affected by the deletion of the *MAT* genes (Figure 8A). Cell wall biogenesis and ROS elimination are vital metabolisms impacting fungal infection. To determine whether the *MAT* genes are involved in cell wall integrity or ROS elimination, the mutants and wild-type strains were incubated on CM containing Congo red, calcofluor white, H_2_O_2_ or methyl viologen. Compared with the wild-type strains, the mutant strains exhibited no significant difference, indicating that the *MAT* genes do not participate in cell wall integrity or ROSs elimination (Figure 8B).

## 4. Discussion

*M. oryzae* has the ability to undergo two modes of reproduction, asexual and sexual, but the latter maybe contribute more to the pathogenicity variation of the fungus and make the rice blast disease difficult to be controlled. Although the natural sexual process of this fungus has not yet been observed in rice fields [15], we found that the field strains have both mating types, and under laboratory conditions, some of these strains can undergo sexual reproduction (data not shown). The investigation of the mechanisms of the sexual cycle in *M. oryzae* may help us to fully understand the life cycle of the destructive pathogen.

Previous studies have investigated the functions of the *MAT* loci in the development of sexual structures in other fungal species. The *MAT* loci are essential for the formation of the sexual structure in *Fusarium graminearum* [17], *Podospora anserine* [10] and *Botrytis cinereal* [18]. As a heterothallic fungus, an *M. oryzae* strain is equipped with one allele of the *MAT* loci, which makes the situation and performance of the *MAT* genes more complicated and difficult to investigate in *M. oryzae* compared with the heterothallic fungal species. In the present work, we investigated the *MAT1-1* and *MAT1-2* loci in *M. oryzae* and revealed their importance in sexual development. This is the first direct evidence that proves the requirement of the *MAT* loci in sex determination and sexual reproduction in *M. oryzae*.

Although *MAT* loci were found to be associated with the proteins that regulate mate recognition, cell fusion and meiosis [10,19,20], the functions and even the numbers of the genes encoded in *MAT* loci vary greatly in different fungal species. *MAT1-1-2* in *F. graminearum* and *MAT1-1-5* in *B. cinerea* are essential in sexual reproduction [18,19,21,22]. In *Sordaria macrospora*, *MAT1-1-2* is a crucial factor for fruiting body and ascospore development, whereas *MAT1-1-1* and *MAT1-1-3* deletion strains showed no difference in sexual reproduction compared to the wild-type strain [19]. *MAT* genes from different species usually have quite low sequence identities, which makes it difficult to deduce the functions of *MAT* genes in a species based on those of others. In the present work, we found that in *M. oryzae*, *MAT1-1-1*, *MAT1-1-3* and *MAT1-2-1* were essential for the development of perithecia, *MAT1-1-2* impacted the formation of asci and ascospores, and *MAT1-2-2* was dispensable for sexual development. The functional differences of *MAT* genes between closely related fungal species indicated the complexity of the regulation of fungal sexual reproduction. The nucleic sequences of *MAT1-1-3* and *MAT1-2-2* in *M. oryzae* are largely identical, while the functions of the two genes differ greatly. The deletion of the former leads to the vanishing of the perithecia, asci and ascospores, while the latter is dispensable for the formation of these structures. Thus, *MAT1-2-2* is likely a redundant version of *MAT1-1-3*, which is perhaps the next topic worthy of investigation. In addition, the subcellular localization of the MAT proteins in *M. oryzae* was also found to be different; MAT1-1-3 was distributed in the nucleus, while MAT1-1-2 and MAT1-2-1 were found to be cytoplasmic. These results are partially the same as those for *F. graminearum* [6].

Sexual reproduction in fungi was regulated by complicated signal transduction networks, such as hormone receptor, G protein and MAPK pathways. Some of the pathways were also deemed as key regulators in the pathogenicity of *M. oryzae* [2,7,10]. In a comparison of the *MAT* loci mutants with the wild-types, however, we found that the *MAT* loci are not involved in vegetative growth, asexual reproduction or pathogenicity. It is maybe an evolutionary adaptation. In most natural environments and for the majority of its life cycle, *M. oryzae* finishes development and reproduction in asexual mode, while the sexual cycle perhaps exists in some specific regions [14,15]. The independence of the regulation of asexual and pathogenic development, with sex determination and sexual reproduction, ensures the highest possibility of survival and pathogenicity of the fungus in environments that are suitable or unsuitable for sexual reproduction. Despite that, how the *MAT* genes trigger or are triggered by the signal pathways, whether the MAT genes function cooperatively or competitively in regulation and whether the regulation networks for sexual reproduction and pathogenicity crosstalk in some way are questions that, to date, are still not fully understood, and more investigations are required in order to find answers.

In addition, the lack of proper methods is a barrier to the study of sexual mechanisms. In recent years, we established a series of related methods for the observation of sexual structures, such as chemical staining and fluorescence labeling [23,24,25]. The proper usage of these methods will be beneficial for the further investigation of the biological, cellular and molecular mechanisms of sexual reproduction in *M. oryzae*.

## 5. Conclusions

In this study, we characterized the roles of the two *MAT* loci and the individual *MAT* genes in *M. oryzae* via gene disruption strategies, provided the direct evidence for the involvement of the *MAT* loci and *MAT* genes in mating type determination and formation of sexual structures, and found the loci and genes are dispersible for pathogenicity and asexual development of the fungus.

## Figures and Tables

**Table 1:** The numbers of perithecia formed in the cross of *MAT* loci mutants, the wild-type strains and complement strains on OMA plates (×10^4^/plate).

	Guy11	TH3	TH16
Guy1		1.3 ± 0.3	
Guy11*^Δmat^*		0	
Guy11*^Δmat1^*^/*MAT*^		1.1 ± 0.1	
TH3	1.3 ± 0.3		
TH3*^Δmat2^*	0		
TH3*^Δmat2^*^/*MAT2*^	1.4 ± 0.2		
70-15			1.0 ± 0.2
70-15*^Δmat2^*			0
70-15*^Δmat2^*^/*MAT2*^			0.9 ± 0.1
2539			1.5 ± 0.3
2539*^Δmat2^*			0
2539*^Δmat2^*^/*MAT2*^			1.5 ± 0.3

**Table 2:** The numbers of perithecia formed in the cross of *MAT* genes mutants, complement strains and the wild-type strains on OMA (×10^4^/plate).

	TH3	TH16	Guy11
Guy11	1.2 ± 0.3		
Guy11^*Δ111*^	0		
Guy11^*Δ112*^	1.2 ± 0.3		
Guy11^*Δ113*^	0		
Guy11^*Δ111/111*^	1.1 ± 0.2		
Guy11^*Δ112/112*^	1.2 ± 0.3		
Guy11^*Δ113/113*^	1.2 ± 0.2		
70-15		1.0 ± 0.3	
70-15^*Δ121*^		0	
70-15^*Δ122*^		1.0 ± 0.2	
70-15^*Δ121/121*^		0.9 ± 0.2	
2539			1.3 ± 0.1
2539^*Δ122*^			1.3 ± 0.3
TH3		1.6 ± 0.3	
TH3^*Δ122*^		1.6 ± 0.4	

## Supplementary Materials

The following are available online at https://www.mdpi.com/article/10.3390/jof7100858/s1, Figure S1 Colonial morphology of wild-type, *MAT* gene mutants and complement strains cultured on CM for 7 days; Table S1. The primers used in this work.
