# Supplementary material for: MAT Loci Play Crucial Roles in Sexual Development but Are Dispensable for Asexual Reproduction and Pathogenicity in Rice Blast Fungus Magnaporthe oryzae
Source: J Fungi (Basel). 2021 Oct 13;7(10):858. doi: 10.3390/jof7100858 (PMC8539793; doi:10.3390/jof7100858)
Supplement: Supplementary file 1 [file jof-07-00858-s001.zip › jof-1362987-supplementary/Table S1.pdf]

Table S1 Primers used in this work

Primer	Sequence（5'-3'）	Function
HKOPB-1-1(1)	ATGTGTTGACCTCCAGGATCCCACATCCATCATACCCCTTCGGGA	MAT loci disruption
HKOPB-1-1(2)	GCCAAGCTTGCATGCCTGCAGCAAGTAGCAGAGATGTGCCATCGG	MAT loci disruption
HKOEX-1-1(1)	ATTATTATGGAGAAACTCGAGGTCGATACAAAGATCCCCCAGAGA	MAT loci disruption
HKOEX-1-1(2)	CCGGGTACCGAGCTCGAATTCTGGAAGCACTTGAGGGAGGCGCCA	MAT loci disruption
HKOPB-1-2(1)	ATGTGTTGACCTCCAGGATCCCTGGTCTCTGGCACTCTGAGCGTG	MAT loci disruption
HKOPB-1-2(2)	GCCAAGCTTGCATGCCTGCAGACCAAAGAAGATGATCAGTCGGCC	MAT loci disruption
HKOEX-1-2(1)	ATTATTATGGAGAAACTCGAGCGGAACACTTACCACATTCGAGTA	MAT loci disruption
HKOEX-1-2(2)	CCGGGTACCGAGCTCGAATTCGTAGATGTGCCTCGTTTCAATGAT	MAT loci disruption
SKOEX1-111	CACGGTACCGAGCTCGAATTCTGGAAGCACTTGAGGGAGGCGCCA	MAT gene disruption
SKOEX2-111	ATTATTATGGAGAAACTCGAGGTCGATACAAAGATCCCCCAGAGA	MAT gene disruption
SKOPB1-111	GCCAAGCTTGCATGCCTGCAGCACCTGAGGCTCATCTGCTGAAGC	MAT gene disruption
SKOPB2-111	CTGTGGCGTTGGCACGGATCCTCAGACCATCTCAGGAGCTCATTT	MAT gene disruption
HKOHB1-112	ACGACGGCCAGTGCCAAGCTTTTCATATCACCCTTTCGTGCACAC	MAT gene disruption
HKOHB2-112	ATGTGTTGACCTCCAGGATCCTCGCGCGCGGCTACCTCTAAATCC	MAT gene disruption
HKOSmK1-112	AAAGGAATAGAGTAGCCCGGGCGCCGTTGACGCGACAGTCATGGA	MAT gene disruption
HKOSmK2-112	TACGAATTCGAGCTCGGTACCCAGAGGTGGGAGATATATGCATGT	MAT gene disruption
SKOPB1-113	GCCAAGCTTGCATGCCTGCAGGCAAGACCATGCATTTGATAGTAG	MAT gene disruption
SKOPB2-113	CTGTGGCGTTGGCACGGATCCTTGTGATATTTGCTGTAAGTGAAG	MAT gene disruption
SKOEX1-113	CACGGTACCGAGCTCGAATTCCACATCCATCATACCCCTTCGGGA	MAT gene disruption
SKOEX2-113	ATTATTATGGAGAAACTCGAGCAAGTAGCAGAGATGTGCCATCGG	MAT gene disruption
HKOPB1-121	GCCAAGCTTGCATGCCTGCAGCGGAACACTTACCACATTCGAGTA	MAT gene disruption
HKOPB2-121	ATGTGTTGACCTCCAGGATCCGTAGATGTGCCTCGTTTCAATGAT	MAT gene disruption
HKOEX1-121	CCGGGTACCGAGCTCGAATTCGAGGTCACCATTCACATCATCCGA	MAT gene disruption
HKOEX2-121	ATTATTATGGAGAAACTCGAGATATATGCATGTCGGAAAGTCCCG	MAT gene disruption
HKOSmX1-122	AAAGGAATAGAGTAGCCCGGGCTGAATATGTTTCGCTGGCAGATA	MAT gene disruption
HKOSmX2-122	ATTATTATGGAGAAACTCGAGTCCCTCCAAGACTCCGACATCACC	MAT gene disruption
HKOPB1-122	GCCAAGCTTGCATGCCTGCAGACCAAAGAAGATGATCAGTCGGCC	MAT gene disruption
HKOPB2-122	ATGTGTTGACCTCCAGGATCCCTGGTCTCTGGCACTCTGAGCGTG	MAT gene disruption
1-1Check2.1	TCCCAGTCACAAATCCCTCACATT	Mutant confirmtion
1-1Check3.1	GTTGCTTCTTCTTCTGCTGCCTCT	Mutant confirmtion

1-1Check2.2	TCCCAGTCACAAATCCCTCACATT	Mutant confirmtion
1-1Check3.2	GCGCCAACTCCTTGATACGTTCTA	Mutant confirmtion
1-1Check2.3	CAGGCTCTGCTCTGTCCACTTCTT	Mutant confirmtion
1-1Check3.3	GATACCATTGGCGTTGATGTTTGT	Mutant confirmtion
1-1Check1	ATCGGAAGGGAGCACAGAAGTGGC	Mutant confirmtion
1-1Check4	CGACTTCCGTCAAGCGTTCCATAA	Mutant confirmtion
1-2Check2.1	GGGTCACGGATTTGTCTGGCTTTG	Mutant confirmtion
1-2Check3.1	GATTTCGCTCGCGTTCATGGATGG	Mutant confirmtion
1-2Check2.2	GGGTCACGGATTTGTCTGGCTTTG	Mutant confirmtion
1-2Check3.2	GCTCCTCCCACTCTGCCTTTGTCG	Mutant confirmtion
1-2Check1	CGAGGCGGTTCCAGGCTGCAAAGA	Mutant confirmtion
1-2Check4	GGGAGACACGCAAGAACGCACGAC	Mutant confirmtion
HPH-UP	TCCTGACGGACAATGGCCGCATAA	Mutant confirmtion
HPH-DN	GGCGCAGCTATTTACCCGCAGGAC	Mutant confirmtion
HPH1	GCTCCATACAAGCCAACC	Mutant confirmtion
HPH2	CTTCTTAAGTTCGCCCTTCC	Mutant confirmtion
RECPB-1-1(1)	GAGCTCGGTACCCGGGGATCCGTCGATACAAAGATCCCCCAGAGA	MAT loci complment
RECPB-1-1(2)	GCCAAGCTTGCATGCCTGCAGCAAGTAGCAGAGATGTGCCATCGG	MAT loci complment
RECPB-1-2(1)	GAGCTCGGTACCCGGGGATCCCGGAACACTTACCACATTCGAGTA	MAT loci complment
RECPB-1-2(2)	GCCAAGCTTGCATGCCTGCAGACCAAAGAAGATGATCAGTCGGCC	MAT loci complment
REC-111(1)	GCCAAGCTTGCATGCCTGCAGCACCTGAGGCTCATCTGCTGAAGCGAT	MAT gene complment
REC-111(2)	GAGCTCGGTACCCGGGGATCCGTCGATACAAAGATCCCCCAGAGACGC	MAT gene complment
REC-112(1)	GCCAAGCTTGCATGCCTGCAGCAGAGGTGGGAGATATATGCATGTCGG	MAT gene complment
REC-112(2)	GAGCTCGGTACCCGGGGATCCTTCATATCACCCTTTCGTGCACACATT	MAT gene complment
REC-113(1)	GCCAAGCTTGCATGCCTGCAGCAAGTAGCAGAGATGTGCCATCGGAAA	MAT gene complment
REC-113(2)	GAGCTCGGTACCCGGGGATCCGCAAGACCATGCATTTGATAGTAGTTG	MAT gene complment
REC-121(1)	GCCAAGCTTGCATGCCTGCAGATATATGCATGTCGGAAAGTCCCGAAG	MAT gene complment
REC-121(2)	GAGCTCGGTACCCGGGGATCCCGGAACACTTACCACATTCGAGTATCC	MAT gene complment
REC-122(1)	GCCAAGCTTGCATGCCTGCAGACCAAAGAAGATGATCAGTCGGCCAAG	MAT gene complment
REC-122(2)	GAGCTCGGTACCCGGGGATCCTCCCTCCAAGACTCCGACATCACCAAA	MAT gene complment
111check 2	GCTGGCATCTTTCAGGATAGACCG	Confirmation of gene complment
111check 3	GTGTTTCCGGGTGACCATGACCTT	Confirmation of gene complment
112check 2	GCAGGTCTAGGACCCGATGAAACA	Confirmation of gene complment
112check 3	GGAACTCTTAGGCGATAGCAGCAT	Confirmation of gene complment

113check 2	TCAATCTGCCACCACAGCGTCCTC	Confirmation of gene complment
113check 3	CCACCTTGTCACCGTCGCCCTTCT	Confirmation of gene complment
121Check2	AGGGTCACGGATTTGTCTG	Confirmation of gene complment
121Check3	TTGCTTTGCTCGGCTTCG	Confirmation of gene complment
122Check2	TGGGTCGTCTGCGTTCCT	Confirmation of gene complment
122Check3	TGCTGCTCCTGTTGCTTC	Confirmation of gene complment
bar1	CCAGAAACCCACGTCATGCCAGTT	Confirmation of gene complment
bar2	AGTCGTCCAGGCGGTGAGCACAAA	Confirmation of gene complment
MAT111-RT-F1	GTTAGCCCGAGAGCGGTAGCA	qRT-PCR assay
MAT111-RT-R1	TCCTGAAAGATGCCAGCGTAG	qRT-PCR assay
MAT112-RT-F1	ATGGAAAACCAGAGCCAGAGGA	qRT-PCR assay
MAT112-RT-R1	CTGCTATCGCCTAAGAGTTCCG	qRT-PCR assay
113 F1	CTACGAGAACAGCATAGTGGCGAG	qRT-PCR assay
113 R1	GATGCCGCAAACTTTGCCTAGCGA	qRT-PCR assay
MAT121-RT-F2	ACAGCACAGAACTTTTCCCAGC	qRT-PCR assay
MAT121-RT-R2	AGGCTCTCATCACCTACCATCG	qRT-PCR assay
MAT122-RT-F2	GCCGCGTTGATTCTCCTCCTCAAT	qRT-PCR assay
MAT122-RT-R2	ACTTGGCGGCACGGTAGAG	qRT-PCR assay
RT-Tubulin-F3	GGCCAATGCGGCAACCAA	qRT-PCR assay
RT-Tubulin-R3	AGGACAGCACGGGGAACA	qRT-PCR assay
