# Supplementary figures and images for: MAT Loci Play Crucial Roles in Sexual Development but Are Dispensable for Asexual Reproduction and Pathogenicity in Rice Blast Fungus Magnaporthe oryzae
Source: J Fungi (Basel). 2021 Oct 13;7(10):858. doi: 10.3390/jof7100858 (PMC8539793; doi:10.3390/jof7100858)

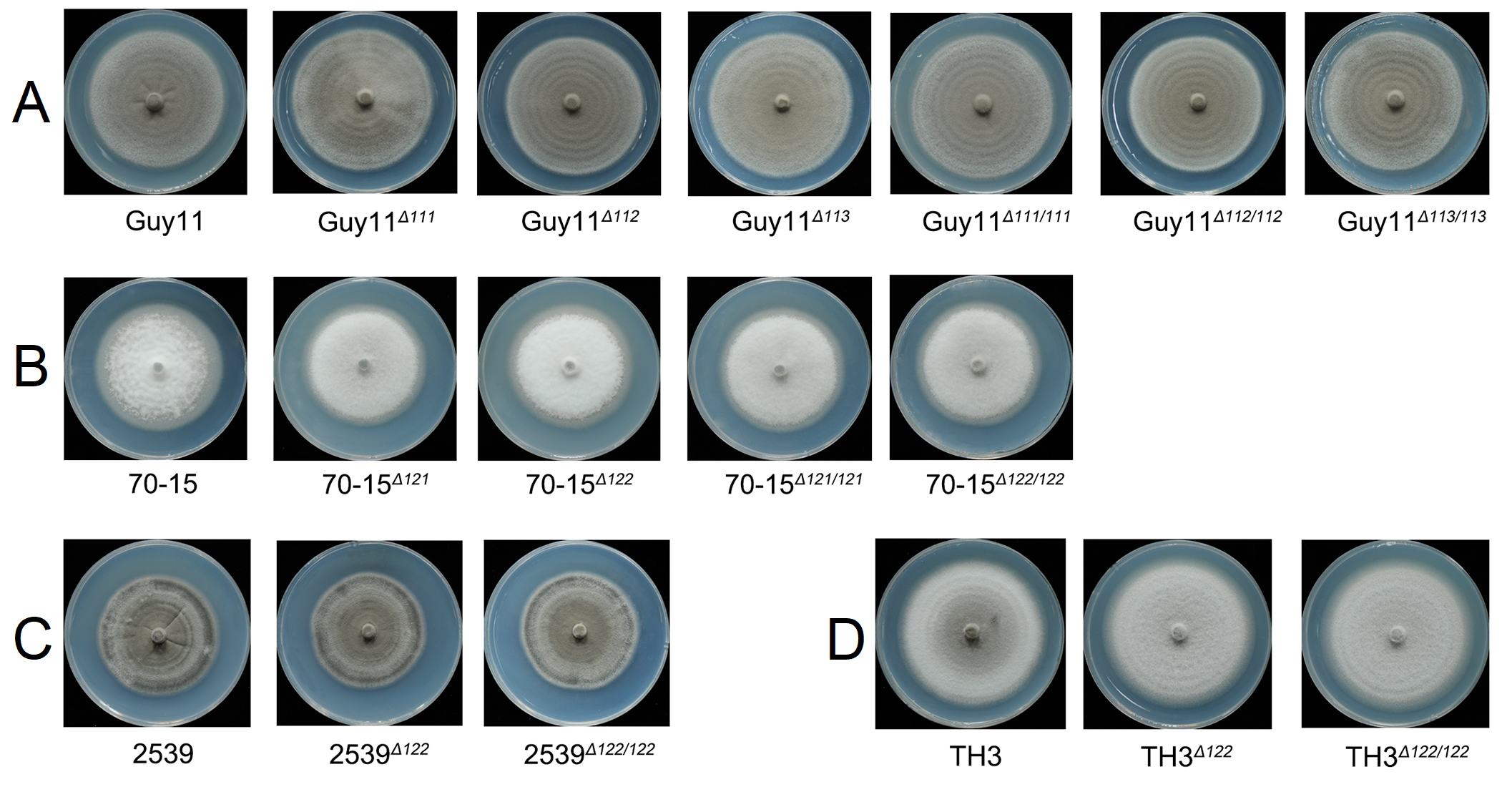

Supplement: Supplementary file 1 [file jof-07-00858-s001.zip › jof-1362987-supplementary/S1.tif]
